# The clinical utility curve: a proposal to improve the translation of information provided by prediction models to clinicians

**DOI:** 10.1186/s13104-016-2028-0

**Published:** 2016-04-14

**Authors:** Duncan J. Campbell

**Affiliations:** Department of Molecular Cardiology, St. Vincent’s Institute of Medical Research and The University of Melbourne, Melbourne, Australia; St. Vincent’s Institute of Medical Research and The University of Melbourne, 41 Victoria Parade, Fitzroy, VIC 3065 Australia

**Keywords:** Prediction models, Clinical decision-making

## Abstract

**Background:**

Prediction models are essential to the development of prediction rules that guide decision-making, and comparison of prediction models with and without an additional diagnostic or prognostic risk factor allows assessment of the value of the additional factor in risk prediction. However, the many different measures described to translate the information provided by a prediction model do not readily assist clinicians’ decision-making.

**Results:**

The clinical utility (CU) curve is proposed as an alternative method of communication of information from a prediction model to the clinician. The CU curve is essentially a derivation of the ROC curve that has sensitivity on the y-axis and the number needed to capture one case (NNCOC) on the x-axis. It provides information about the relationship between sensitivity and false positive rate over the full range of prediction score thresholds, and it also indicates the proportion of the patient population with an absolute risk below the threshold for 100 % sensitivity that can therefore be classified as free of disease.

**Conclusions:**

The CU curve is proposed as a means to assist the translation of model information to the clinician, in the hope that it will stimulate debate and, through refinement, assist the development of prediction rules with optimal clinical utility.

**Electronic supplementary material:**

The online version of this article (doi:10.1186/s13104-016-2028-0) contains supplementary material, which is available to authorized users.

## Background

Prediction models are essential to inform healthcare professionals and individuals about the risks of having (diagnosis) or developing (prognosis) a particular disease or outcome. This information guides clinicians’ decision-making regarding further management—including additional testing and initiating or withholding treatment(s) and lifestyle changes [1]. Moreover, comparison of prediction models with and without an additional diagnostic or prognostic risk factor allows assessment of the value of the additional factor in risk prediction. The challenge for clinicians is to translate the prediction model to a prediction rule, which requires the definition of a prediction score cut-off or threshold [2]. Translation takes a number of factors into account, including the sensitivity, specificity, and false positive rate of a particular prediction score threshold, and the balance between the potential benefits and costs that result from a prediction rule based on the threshold (see Additional file 1: Glossary).

Many different measures have been described to translate the information provided by a prediction model to decision-making, and for the assessment of the incremental value of additional risk factors. These include the use of odds ratios to assess the association of a risk factor with a clinical outcome, R squared, Brier score and integrated discrimination improvement (IDI) indices to assess overall performance of the prediction model, receiver operating characteristic (ROC) curves and C statistics to assess how well the model discriminates between patients with and without the disease or outcome, net reclassification improvement (NRI) to assess whether a new model improves patient classification, and net benefit, weighted NRI, decision curve analysis, clinical utility index, and relative utility curves to assess the clinical utility of the model [3–6].

These measures for translating information provided by prediction models do not readily provide the information that clinicians require for decision-making, and there is ongoing debate about the appropriateness of some of these measures [7, 8]. As discussed by Steyerberg et al. these measures may have limitations with respect to specific prediction score thresholds because different thresholds can offer different conclusions about the relative merits of different prediction models [4], and a substantial change in any of these parameters achieved by adding a risk factor to a model may not translate to clinical usefulness at a given threshold [4, 9]. Although C statistics, NRI and IDI may show that a prediction model has superior performance, they do not convey information about the consequences of the improved performance. Net benefit, weighted NRI, clinical utility index, relative utility curves, and decision curve analysis may appear to offer advantages [4], but a clinical decision about weighting is required before the modeler can calculate these parameters. For example, it is the clinician who decides how many biopsies of people without cancer (and the potential harm from biopsy) justifies one correctly diagnosed case of cancer. This clinical decision may vary depending on the clinical context, including the patient’s age, sex and co-morbidities.

Critical information required by the clinician for decision-making includes the sensitivity for detection of cases or future cases by a particular prediction score threshold, and the corresponding number of false positives captured by this threshold. Negative predictive value is also of importance for the clinician who wants to “rule out” a diagnosis or risk. The objective of this manuscript is to describe the clinical utility (CU) curve. The CU curve is a proposal to improve the translation of information provided by prediction models to clinicians. This is essentially a derivation of the ROC curve that has sensitivity on the y-axis and the number needed to capture one case (NNCOC) on the x-axis for the full range of prediction score thresholds. For example, if a prediction rule is to help decide how many patients to biopsy to confirm a diagnosis of cancer, the NNCOC is the number of patients that need to be biopsied to diagnose one case of cancer. The CU curve therefore provides information about the number of false positives for each true positive, in relation to sensitivity. The NNCOC (the reciprocal of positive predictive value) is analogous to the number needed to treat, which is the reciprocal of absolute risk reduction by a therapeutic intervention. Thus, for patients with high blood pressure, the number needed to treat refers to the number who need to be treated with an antihypertensive agent in order to prevent one cardiovascular event [10]. The novelty of the CU curve is that it allows the clinician to explore the interplay between sensitivity and false positive rate over a range of prediction score thresholds. When taken into account with the cost of the test and its potential harm, the CU curve assists the clinician in assessing misclassification costs and harm-benefit ratio that apply to any particular threshold, and in thereby determining which prediction score threshold results in a prediction rule with optimal clinical utility.

## Methods

Two examples of the CU curve are presented. These are for illustrative purposes only, based on publicly available data [11, 12], and are focused on dichotomous outcomes. Absolute risk for each patient was calculated by performing logistic regression of each multivariable model using the PredictABEL package in R. NRI and IDI were also computed with the PredictABEL package. C statistics were calculated using the pROC package in R. The CU curve for a model was constructed by entering each patient’s absolute risk determined from the model into an Excel spreadsheet together with data about the presence or absence of malignancy (example 1) or coronary disease (example 2). The patients were sorted according to their absolute risk, then sensitivity, specificity, NNCOC and other parameters calculated for each patient.

## Results

### Example 1: assessment of the value of a new biomarker

For women with adnexal masses, the clinician wants to identify all cases of malignancy. It is also an advantage to identify women without malignancy before surgery because a simpler surgical procedure may be performed in these women. Carter et al. reported a series of 37 women with adnexal masses, of which 12 were malignant and three had borderline histology (herein combined and referred to as malignant) [11]. These authors examined the predictive ability of quantitative T_2_ mapping of contrast-enhanced imaging of the masses identified by magnetic resonance imaging. In the present analysis, a prediction model based on age, largest dimension of the mass encompassing all regions of interest and number of solid regions of interest per mass (model 1) was compared with a model based on the same 3 parameters plus the number of cystic regions of interest per mass (model 2). Both models were well calibrated (Hosmer–Lemeshow test: P = 0.405 for model 1; P = 0.413 for model 2). The C statistic for model 1 was 0.839 (95 % CI 0.697–0.947) and for model 2 was 0.877 (0.753–0.968), P = 0.003 (bootstrap estimate). Comparing models 1 and 2, the NRI (continuous) was 0.74 (0.14–1.34), P = 0.016, and the IDI was 0.072 (−0.019 to 0.163), P = 0.120.

**Fig. 1:**
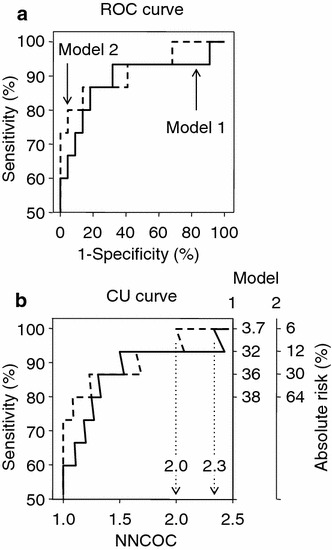
ROC curves (**a**) and CU curves (**b**) for the prediction of malignancy (combined malignancy and borderline histology) in 37 women with adnexal masses. *Solid line* represents model 1 and *dashed line* represents model 2. *NNCOC* number of surgical biopsies needed to capture one case of malignancy. C statistics were 0.839 (95 % CI 0.697–0.947) for model 1 and 0.877 (0.753–0.968) for model 2, P = 0.003. Sensitivities of 80, 87, 93 and 100 % were achieved at absolute risks of 38, 36, 32 and 3.7 %, respectively, for model 1, and 64, 30, 12 and 6 %, respectively for model 2. Model 1 identified all 15 malignancies in 35 patients, with an NNCOC of 2.3, whereas model 2 identified all 15 malignancies in 30 patients, with an NNCOC of 2

The CU curves resemble the respective ROC curves when the models are applied to everyone in the dataset, except that the x-axis is NNCOC rather than 1-specificity (Fig. 1). This is to be expected because 1-specificity is the same as the false positive rate (expressed as a percentage). The CU curves give the clinician easily understood information about the number of surgical biopsies required to identify one case of malignancy according to the proportion of malignancies identified (sensitivity) for each model, and the number of patients identified without malignancy, whereas the ROC curves do not convey this information. The top right-hand corner of the CU curve corresponds to the NNCOC achieved when all members of the population are biopsied, and is always at 100 % sensitivity because all malignancies are identified if all patients are biopsied. Where the curve reaches 100 % sensitivity before all patients are biopsied, the length of the horizontal line at 100 % sensitivity indicates the proportion of the patient population with a prediction score below the threshold for 100 % sensitivity, which can therefore be identified as free of malignancy before surgery and biopsy.

The CU curve for model 1 shows that it was necessary to biopsy 35 patients with an absolute risk ≥3.7 % to identify the 15 with malignancy, producing an NNCOC of 2.3 when the CU curve first reached 100 % sensitivity; model 1 identified only 2 patients without malignancy. By contrast, model 2 identified all 15 patients with malignancy among 30 patients with an absolute risk ≥6 %; thus, the NNCOC was 2 when the CU curve first reached 100 % sensitivity, and model 2 identified 7 patients without malignancy. Different absolute risks were required to identify malignancy with the same sensitivity with models 1 and 2. For example, an absolute risk ≥32 % identified 93 % of malignancies using model 1, whereas a risk ≥12 % was required to identify 93 % of malignancies using model 2 (Fig. 1).

### Example 2: comparison of patient subgroups using a multivariable prediction model

The prediction of coronary artery disease in men and women was compared using a prediction model based on a dataset of 303 patients referred to the Cleveland Clinic for coronary angiography [12]. None had a history or electrocardiographic evidence for prior myocardial infarction or known valvular or cardiomyopathic disease. There were 206 men and 97 women; 114 men and 25 women had one or more coronary arteries with >50 % stenosis. The prediction model was constructed using all 303 patients, based on age, sex, character of chest pain, resting systolic blood pressure on admission, serum cholesterol level, fasting blood glucose >120 mg/dl, resting electrocardiograph, maximum heart rate achieved during exercise, exercise-induced angina, ST segment depression during exercise, slope of the ST segment during peak exercise, number of calcified vessels detected by fluoroscopy, and the result of an exercise thallium scintigraphy test. The outcome was patients with ≥1 coronary arteries with >50 % stenosis. The model was well calibrated (Hosmer–Lemeshow test: P = 0.367) and the C statistic was 0.873 (0.833–0.913) (Delong method) [13]. Male sex was a significant predictor in the multivariable model with a coefficient of 1.98 (SE 0.38).

**Fig. 2:**
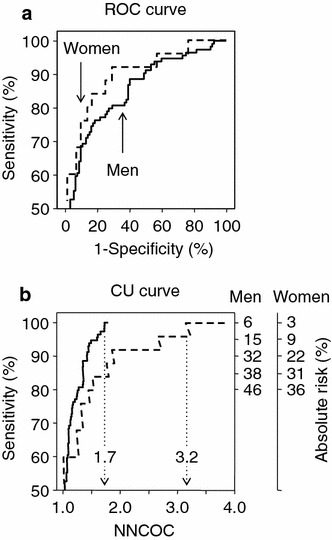
ROC curves (**a**) and CU curves (**b**) for the prediction of patients with ≥1 coronary arteries with >50 % stenosis. *Solid line* represents men (n = 206) and *dashed line* represents women (n = 97). NNCOC, number needed to capture one patient with ≥1 coronary arteries with >50 % stenosis. C statistics were 0.846 (95 % CI 0.792–0.900) for men and 0.892 (0.812–0.973) for women, P = 0.348. The model achieved sensitivities of 80, 85, 90, 95 and 100 % at absolute risks of 46, 38, 32, 15 and 6 %, respectively, for men, and 36, 31, 22, 9 and 3 %, respectively, for women. The model identified all 114 men with ≥1 coronary arteries with >50 % stenosis in 199 men who underwent coronary angiography, with an NNCOC of 1.7, whereas when applied to women, the model identified all 25 women with ≥1 coronary arteries with >50 % stenosis in 80 women who underwent coronary angiography, with an NNCOC of 3.2

The ROC curves were similar for men and women, with C statistics of 0.846 (0.792–0.900) for men and 0.892 (0.812–0.973) for women, P = 0.348 (Delong method) [13] (Fig. 2). However, despite the similarity in ROC curves, the CU curves show the prediction model performed differently in men and women. The model identified all 114 men with ≥1 coronary arteries with >50 % stenosis in 199 men who underwent coronary angiography, with an NNCOC of 1.7 whereas, when applied to women, the model identified all 25 women with ≥1 coronary arteries with >50 % stenosis in 80 women who underwent coronary angiography, with an NNCOC of 3.2. Different absolute risks were required to identify men and women with ≥1 coronary arteries with >50 % stenosis with the same sensitivity. For example, an absolute risk ≥32 % identified 90 % of men, whereas an absolute risk ≥26 % identified 90 % of women with coronary artery disease (Fig. 2). Despite the higher NNCOC for women, it is evident that the model was better able to exclude coronary artery disease in women than in men. An absolute risk threshold of 6 % identified only 7 of 92 men without coronary artery disease, whereas a threshold of 3 % identified 17 of 72 women without coronary artery disease, who may have avoided coronary angiography.

## Discussion

These two examples indicate how the CU curve may improve the translation of information provided by prediction models to clinicians to assist the development of prediction rules by directly comparing two models with and without an additional risk factor, and by directly comparing the performance of a risk model in different patient subgroups. The CU curve is based on the same information used to calculate the ROC curve, but it provides a more complete description of this information than the ROC curve. The CU curve provides information about the relationship between sensitivity and false positive rate over a range of prediction score thresholds, and also indicates the proportion of the patient population with an absolute risk below the threshold for 100 % sensitivity that can therefore be identified as free of disease. The CU curve assists clinicians in assessing the tradeoff between sensitivity and false positive rate, and in applying their own weighting when they interpret the data.

Going from a prediction model to a prediction rule requires the definition of a prediction score threshold [2]. Current strategies for comparing prediction models are not intuitively easy for the clinician to use when deciding whether one model is superior to another or when choosing a prediction score threshold to use in a prediction rule. Steyerberg et al. recommends that the decision-analytic measure net benefit or weighted NRI is preferable for the identification of the most informative prediction model and the prediction score threshold that provides greatest clinical utility [4]. Net benefit is a measure that explicitly incorporates the relative weight for over-diagnosis vs. appropriate diagnosis [14, 15]. However, the clinician, not the modeler, decides weighting, and net benefit, weighted NRI, clinical utility index, relative utility curves, and decision curve analysis require that the clinician has already made a decision about weighting before they can be calculated. Another limitation of net benefit, weighted NRI, decision curve analysis, clinical utility index and relative utility curves is that they do not report sensitivity, which is an important consideration for the clinician who wants a prediction rule that benefits the greatest number of individuals.

When managing an individual patient, most physicians implicitly consider the tradeoffs that influence the decision whether to administer or withhold therapy, or order another test. The CU curve is different from alternative methods of assessment of diagnostic or prognostic markers in that it helps the clinician assess the interplay between sensitivity, false positive rate and prediction score threshold without the need for additional calculations. It also allows assessment of improvement in classification because higher sensitivity will reduce false-negatives, and lower NNCOC will reduce false positives. By providing a more intuitive comparison of prediction models the CU curve assists the clinician in weighing the potential costs, including harm, and benefits of an additional diagnostic or prognostic marker. If a new marker reduces the false positive rate for the same prediction score threshold, then the clinician may choose to lower the threshold to capture of a greater proportion of cases or future cases while maintaining the false-positive rate.

The prediction score threshold for a prediction rule should take account of the clinical context and the consequences of a clinical decision based on the prediction rule [16]. If cost is a critical factor, then a clinician working with fewer cost constraints may choose a different prediction score threshold than a clinician working with more cost constraints. If potential harm, such as from diagnostic investigation, is a critical factor, then a clinician with access to skills and technology may choose a different prediction score threshold than a clinician without access to these resources. The CU curve allows the clinician to choose a prediction score threshold appropriate to their circumstance-specific weighting for detection of disease vs. investigating patients without disease.

Multivariable prediction models such as the Framingham and Pooled Cohort Equations for cardiovascular risk prediction are applied to populations comprising men and women of different ages [17, 18]. However, an important concern for the clinician is whether these models perform similarly for different patient subgroups. The ROC curve may indicate a test with high discrimination, but it does not indicate how well the test will perform in different populations with different disease prevalence [19]. By contrast, the CU curve illustrates how the test performs in different populations such as men compared with women. By allowing the direct comparison of CU curves for patient subgroups, the CU curve assists the clinician in determining the optimal prediction score threshold for each subgroup. The CU curves for men and women of the coronary artery disease cohort in example 2 showed that the prediction model performed differently for men and women, despite the prediction model algorithm including sex as a predictor. The multivariable prediction model of the Pooled Cohort Equation similarly failed to adequately account for differences between subgroups, as shown by the differences between men and women and between different age groups in the prediction of cardiovascular risk [20]. The clinician is more likely to ask the modeler to create separate subgroup-specific prediction models if they are aware of the different performance of a risk model for different patient subgroups.

## Limitations

Like all measures for the translation of information from prediction models to prediction rules, the CU curve has limitations. It fails to give direct information about the negative predictive value for a model. However, a key determinant of the negative predictive value is the false-positive rate, and it is apparent that the negative predictive value can be maximized by keeping sensitivity as high as possible (and false negatives as low as possible). The figure depicting the CU curve will need to be adapted to the model. In the examples shown in Figs. 1 and 2 it was possible to show NNCOC in absolute units. However, for a model with a small positive predictive value, the NNCOC may be correspondingly large, and it may be necessary to truncate the x-axis or to log transform NNCOC so that the CU curve can most effectively translate information about the model to the clinician. It is also evident from examples 1 and 2 that CU curves can be seen to zigzag, in contrast to ROC curves, because the NNCOC is not necessarily a monotonic function of absolute risk or sensitivity. The NNCOC, the reciprocal of positive predictive value, is calculated from the ratio of (true positive plus false positive cases)/true positive cases. There is therefore a stepwise incremental change in NNCOC determined by the changes in the numbers of cases in the numerator and denominator of this relationship, which may cause the CU curve to zigzag. However, this imperfection of the CU curve does not detract from its ability to translate model information to the clinician. Other limitations relate to over-fitting of the CU curve, sampling variability and the confidence intervals for the NNCOC. Although a bootstrap resampling procedure may help to estimate confidence intervals, it would be more appropriate to validate the CU curve in separate patient populations.

## Conclusions

In conclusion, the CU curve is proposed as a means to assist the translation of model information to the clinician, in the hope that it will stimulate debate and, through refinement, assist the development of prediction rules with optimal clinical utility.

## Additional file

10.1186/s13104-016-2028-0 Glossary of terms.

## Abbreviations

CU curve: clinical utility curve
IDI: integrated discrimination improvement
NNCOC: number needed to capture one case
NRI: net reclassification improvement
ROC curve: receiver operating characteristic curve
