# Supplementary material for: The clinical utility curve: a proposal to improve the translation of information provided by prediction models to clinicians
Source: BMC Res Notes. 2016 Apr 14;9:219. doi: 10.1186/s13104-016-2028-0 (PMC4832451; doi:10.1186/s13104-016-2028-0)
Supplement: Supplementary file 1 — 10.1186/s13104-016-2028-0 Glossary of terms. [file 13104_2016_2028_MOESM1_ESM.docx]

**Glossary**

Absolute risk: The probability that an individual will have (diagnosis) or develop (prognosis) a particular disease or outcome, as calculated by the prediction model.

C statistic: The area under the receiver operating curve.

Clinical utility curve: A plot of sensitivity against the number needed to capture one case (reciprocal of the positive predictive value).

False negative rate: The proportion of individuals that have or will develop a disease or outcome that have an absolute risk below the prediction score threshold, and are therefore incorrectly classified as "not at risk".

False positive rate: The proportion of individuals that do not have or will not develop a disease or outcome that have an absolute risk above the prediction score threshold, and are therefore incorrectly classified as "at risk".

Integrated discrimination improvement:

The difference in discrimination slopes between two prediction models, where the discrimination slope of a model is the difference in mean predicted probabilities of disease in individuals classified by the model as having or not having disease.

Negative predictive value: The proportion of individuals with an absolute risk below the prediction score threshold, and therefore classified as "not at risk", that does not experience the disease or outcome.

Net reclassification improvement:

The sum of the net improvement in classification of individuals with events (expressed as a proportion of the number with events) and the net improvement in classification of individuals without events (expressed as a proportion of the number without events).

Positive predictive value: The proportion of individuals with an absolute risk above the prediction score threshold, and therefore classified "at risk", that has or will develop the disease or outcome.

Prediction score threshold: The absolute risk that divides individuals into those "at risk" to have (diagnosis) or develop (prognosis) a particular disease or outcome, and those "not at risk".

Receiver operating curve: A plot of sensitivity against the false-positive rate (1-specificity).

Sensitivity: The proportion of individuals who have or will develop a disease or outcome that is correctly classified by the prediction score threshold to be "at risk".

Specificity: The proportion of individuals who do not have or will not develop a disease or outcome that is correctly classified by the prediction score threshold as "not at risk".
